# Role of Biosensing Technology for NeuroAIDS Management

**DOI:** 10.4172/2155-6210.1000e141

**Published:** 2016-02-29

**Authors:** RD Jayant, M Nair

**Affiliations:** Center of Personalized Nanomedicine, Institute of Neuro Immune Pharmacology, Department of Immunology, Herbert Wertheim College of Medicine, Florida International University, Miami, FL-33199, USA

## Editorial

Effective prevention of HIV/AIDS needs timely diagnosis, introduction of therapy, routine plasma viral load monitoring; viral rebound assessment by precise and sensitive assays for desirable therapeutic manipulations [1]. Because of high costs and time consuming HIV tests, new ways in diagnosis, that offer rapid, cheap, and accurate results are highly desirable. In the past decades, we have experienced massive scientific and technical developments to achieve simple, cost-effective, and rapid diagnostic tests for HIV detection [2]. Among other organs, CNS serve as potent viral reservoirs and therefore monitoring of HIV-1 at CNS level is crucial for early diagnosis and development of subsequent treatment strategies for neuroAIDS treatment. Qualitative and quantitative strategies have been used for the diagnostics of HIV-1, staging of HIV-1, progression and selection of ARV therapy.

Currently biomarkers of CNS diseases are being used to study the progression of HIV as the brain and spinal cord cannot be assessed due to its inaccessibility. Diagnostic tests have focused on HIV-1-associated biomarkers including the presence of host cell integrated proviral DNA and the viral capsid protein antigen p24 [3]. Unfortunately, biomarkers with high accuracy and measureable assessments have not been found due to high genetic variability and wide heterogeneity related with HIV-1. Due to these restrictions, an amalgamation of different markers, multi-marker assays or methods are needed to predict the HIV-1 disease progression and therapeutic effects [4,5]. Presently, CD4+ cell count and RNA viral loads are the two most commonly used analytical markers in the clinical assessment of HIV infection and disease progression [6,7]. However, these markers have variable predictive values that depend on which stage the disease is in and cannot explain all differences of disease progression. In order to monitor progress, which is supposed to aid as the substrate for neuropathology in HIV Associated Neurocognitive Disorders (HAND), soluble cerebrospinal fluid (CSF) markers of macrophage activation (neopterin), chemokines stimulators of macrophages and lymphocytes and molecules involved at various phases in the pathways for cell turnover and activation within the CNS compartment are used [8]. However, immune activation for HIV infection lacks specificity, despite moderately high levels of the previous markers have been correlated to disease activity, they have not been clinically used for diagnosis or monitoring of neuroAIDS.

Point-of-care technologies and other novel detection assays are increasingly used to access to HIV monitoring services in the developing countries. These technologies are endurable, portable, easy to use, and provide acceptable accuracy in ARV therapy [9]. One of the most promising fields is electrochemistry, which is providing innovations with very high specificity, reasonably low prices, and the possibility of miniaturization. Good sensitivity of biosensing methods also help the detection of HIV virions during the diagnostic window, i.e. the period when HIV antibodies are produced, but are under detection limits of common diagnostic methods [10]. In recent years, various successful studies have been performed to identify HIV using electrochemical biosensors for HIV sequence detection e.g. covalently immobilized HIV probe for single-strand DNA (ssDNA) on the modified glassy carbon electrode (GCE) using Aquabis (1,10-phenanthroline) copper(II) Perchlorate [11], bio-organometallic approach for detection of HIV-1 protease (HIV-1 PR) using surface bound ferrocenoyl (Fc)-pepstatin conjugates, or nano-MnO_2_/chitosan composite film modified glassy carbon electrode (MnO_2_/CHIT/GCE) and immobilized DNA probe on the electrode surface [12,13]. Picomolar (pM) level detection of HIV type-1 protease (HIV-1 PR) using ferrocene (Fc)-pepstatin-modified surfaces have also been achieved via electrochemical approach [14]. Recently, aminated diamond-based RNA aptasensor for HIV transactivator of transcription (Tat) peptide-protein has been investigated for HIV detection [15]. Furthermore, there have been HIV biosensing studies involving hybrid Fe_3_O_4_@SiO_2_ nanomagnetic probes and nanogold colloid-labeled enzyme-antibody copolymer [16]. Additionally, polyelectrolyte based magneto-gold nanoparticle based immune sensors have been used to detect the HIV p24 in serum more effectively compared to standard p24 ELSIA technique.

Biosensors for detection peptide and/or protein of HIV showed more advantages over conventional methods for detection and/or identification of the virus. Conventional immunoassay procedures need enzyme or fluorescent-labelled antibody/antigen, lengthy analysis, highly skilled personnel, specially equipped laboratories, and expensive chemicals. On the other hand, the use of above mentioned electrochemical hybridization biomarkers detection could provide a simple and rapid detection and might have a promising future in transducing DNA hybridization and diagnosis for HIV and AIDS treatment. Recent efforts in diagnostics development for the identification and/or detection of HIV are beginning to produce solutions that could be used in the low-resource setting for developing countries. In particular, these electrochemical methods have the potential to be affordable for even the lowest-resource settings. Moreover, substantial efforts are being made to develop novel sensing technologies, novel signaling transduction, and imaging pathways to monitor HIV infection progression. Nano enable sensing assays and biosensor have been used for screening and detection HIV markers at picomolar (pM) level. At clinical level, such investigated nanoenabling sensing systems technologies integrated with drug delivery systems can be promoted for the treatment of neuroAIDS. [17]. Therefore, electrochemical sensors methods can be used as a novel tool for diagnostic, prognostic and therapeutic applications in our effort to control the HIV and neuro-AIDS.
